# Comparison analysis of the triglyceride-glucose index, triglyceride-glucose and frailty index in cardiovascular disease and mortality association among patients with rheumatoid arthritis: A population-based cohort study

**DOI:** 10.1097/MD.0000000000051143

**Published:** 2026-10-16

**Authors:** Yangyu Xu, Hong Zhao, Yuxiang Gao, Jiannan Han, Zewen Wu, Liyun Zhang, Junkang Zhao

**Affiliations:** a Third Hospital of Shanxi Medical University, Shanxi Bethune Hospital, Shanxi Academy of Medical Sciences, Tongji Shanxi Hospital, Taiyuan, Shanxi Province, China; b School of Public Health, Shanxi Medical University, Taiyuan, Shanxi Province, China; c Shanxi Province Clinical Research Center for Dermatologic and Immunologic Diseases (Rheumatic Diseases), Taiyuan, Shanxi Province, China.

**Keywords:** cardiovascular disease, cardiovascular mortality, frailty index, rheumatoid arthritis, triglyceride-glucose index

## Abstract

Cardiovascular disease (CVD) is a major cause of excess mortality among patients with rheumatoid arthritis (RA). Conventional risk assessment may not fully capture the metabolic abnormalities and physiological vulnerability associated with RA. We investigated whether the triglyceride-glucose–frailty index (TyG-FI), which combines a surrogate marker of insulin resistance with accumulated health deficits, was associated with prevalent CVD and cardiovascular mortality among adults with RA. We analyzed 1090 adults with self-reported RA from 10 cycles of the National Health and Nutrition Examination Survey conducted between 1999 and 2018. TyG-FI was calculated as the product of the triglyceride-glucose index and a 49-item deficit-accumulation frailty index. Multivariable logistic regression was used to examine the association with prevalent CVD, whereas Cox proportional hazards regression was used to examine cardiovascular mortality. Restricted cubic splines, subgroup analyses, sensitivity analyses, and discrimination analyses were conducted as secondary analyses. In the fully adjusted models, each 1-unit higher TyG-FI was associated with higher odds of prevalent CVD (odds ratio, 4.50; 95% confidence interval, 3.20–6.33) and a higher hazard of cardiovascular mortality (hazard ratio, 1.77; 95% confidence interval, 1.16–2.70). The reported areas under the receiver operating characteristic curve were 0.80 for prevalent CVD and 0.81 for cardiovascular mortality in models incorporating TyG-FI, compared with 0.73 and 0.80, respectively, in models incorporating TyG. An exploratory subgroup analysis suggested a stronger association between TyG-FI and prevalent CVD among participants aged ≤65 years (*P* for interaction = .044). Higher TyG-FI was associated with prevalent CVD and cardiovascular mortality among US adults with self-reported RA. TyG-FI may capture complementary metabolic and frailty-related information; however, its incremental predictive value, calibration, clinical utility, and external validity require further evaluation before clinical implementation.

**Key Points**

**What is currently known about this topic**

Rheumatoid arthritis patients have a significantly higher risk of cardiovascular disease than the general population.

The TyG index is a simple alternative indicator of insulin resistance and is closely related to cardiovascular prognosis.

**What is the key research question**

By comparing and analyzing TyG and TyG-FI, observe which is better at discriminating cardiovascular disease and mortality in patients with rheumatoid arthritis.

**What is new**

The TyG-FI has significantly better discriminative ability for cardiovascular disease and mortality rates in patients with rheumatoid arthritis compared with the TyG single indicator.

**How might this study influence clinical practice**

TyG-FI may help identify patients with RA who have a greater combined burden of metabolic dysfunction and frailty and who may warrant comprehensive cardiovascular assessment.

## 1. Introduction

Rheumatoid arthritis (RA) is a systemic autoimmune disease characterized by chronic synovial inflammation and progressive joint damage.^[1]^ Cardiovascular disease (CVD) is a major cause of excess morbidity and mortality among patients with RA, whose cardiovascular risk is higher than that of the general population.^[2–4]^ Chronic systemic inflammation contributes to endothelial dysfunction and atherosclerosis in RA.^[2,5]^

Several approaches have been used to estimate cardiovascular risk in RA. General-population algorithms evaluated in this setting include the Framingham Risk Score, Systematic Coronary Risk Evaluation, Reynolds Risk Score, and the American College of Cardiology/American Heart Association Pooled Cohort Equations. These models rely mainly on conventional risk factors and may be miscalibrated in RA populations.^[6,7]^ The 2015/2016 European Alliance of Associations for Rheumatology recommendations advised applying a 1.5 multiplier to algorithms that do not already account for RA.^[2]^ QRISK3 was developed for the general population but includes RA and corticosteroid use.^[8]^ The Expanded Cardiovascular Risk Prediction Score for Rheumatoid Arthritis combines conventional predictors with RA-specific measures, including disease activity, functional disability, prednisone use, and disease duration.^[9]^ External validation has shown variable performance, and RA-adapted or RA-specific tools have not consistently outperformed general-population models.^[7,10]^

The triglyceride-glucose (TyG) index, calculated from fasting triglyceride and glucose concentrations, is an accessible surrogate marker of insulin resistance (IR).^[11,12]^ IR is associated with metabolic syndrome, endothelial dysfunction, and CVD.^[13]^ The TyG index represents a single metabolic domain and does not capture the broader reduction in physiological reserve that may accompany RA.^[11,14]^

The frailty index (FI) quantifies accumulated health deficits across functional, psychological, clinical, and physiological domains.^[14]^ Frailty is more prevalent among patients with RA and is associated with adverse outcomes and mortality.^[15,16]^ The TyG index and FI capture distinct but potentially complementary dimensions of cardiovascular vulnerability.^[11,14]^ Triglyceride-glucose–frailty index (TyG-FI) was selected to integrate metabolic dysfunction with accumulated health deficits, not as a presumed replacement for established cardiovascular risk models.^[17]^ A previous population-based study reported associations of TyG-FI with CVD and stroke, providing an empirical basis for examining this composite index in adults with RA.^[17]^

The associations of TyG-FI with prevalent CVD and cardiovascular mortality in adults with RA remain unclear. Using data from the National Health and Nutrition Examination Survey (NHANES), we examined the cross-sectional association between TyG-FI and prevalent CVD and the longitudinal association between TyG-FI and cardiovascular mortality. We also compared the discriminative ability of models incorporating TyG-FI with that of models incorporating the TyG index alone.

## 2. Materials and methods

### 2.1. Data sources

The NHANES is a national cross-sectional surveillance project conducted by the National Center for Health Statistics, a component of the Centers for Disease Control and Prevention. The project employs a multistage stratified probability sampling design to systematically collect multidimensional health information from the general US population and noninstitutionalized civilians. Through standardized questionnaires, clinical examinations, and laboratory tests, it constructs a comprehensive database, aiming to provide core scientific evidence for assessing the health and nutritional status of the US population. NHANES adheres to strict ethical guidelines throughout its process: the research protocol has been approved by the National Center for Health Statistics Ethics Review Committee, and all participants have signed written informed consent forms. The design and reporting of this study strictly follow the Strengthening the Reporting of Observational Studies in Epidemiology guidelines. Detailed methods and data for the project can be accessed through the official platform: https://www.cdc.gov/nchs/nhanes/.

### 2.2. Study population

We used data from 10 NHANES cycles conducted between 1999 and 2018. Eligible participants were adults aged ≥18 years who were classified as having RA according to the NHANES arthritis questionnaire. RA was identified using participants’ responses to “Which type of arthritis was it?” Participants who reported another or an unspecified type of arthritis were excluded.

Of the NHANES participants assessed during the study period, we sequentially excluded those aged <18 years (n = 42,112), those not classified as having RA (n = 56,252), those with missing triglyceride, fasting glucose, or frailty-index data required to calculate TyG-FI (n = 1743), those with missing CVD information (n = 6), and those with missing prespecified covariates or mortality information (n = 113). The final analytical sample comprised 1090 participants. The participant-selection process is presented in Figure 1.

**Figure 1.**
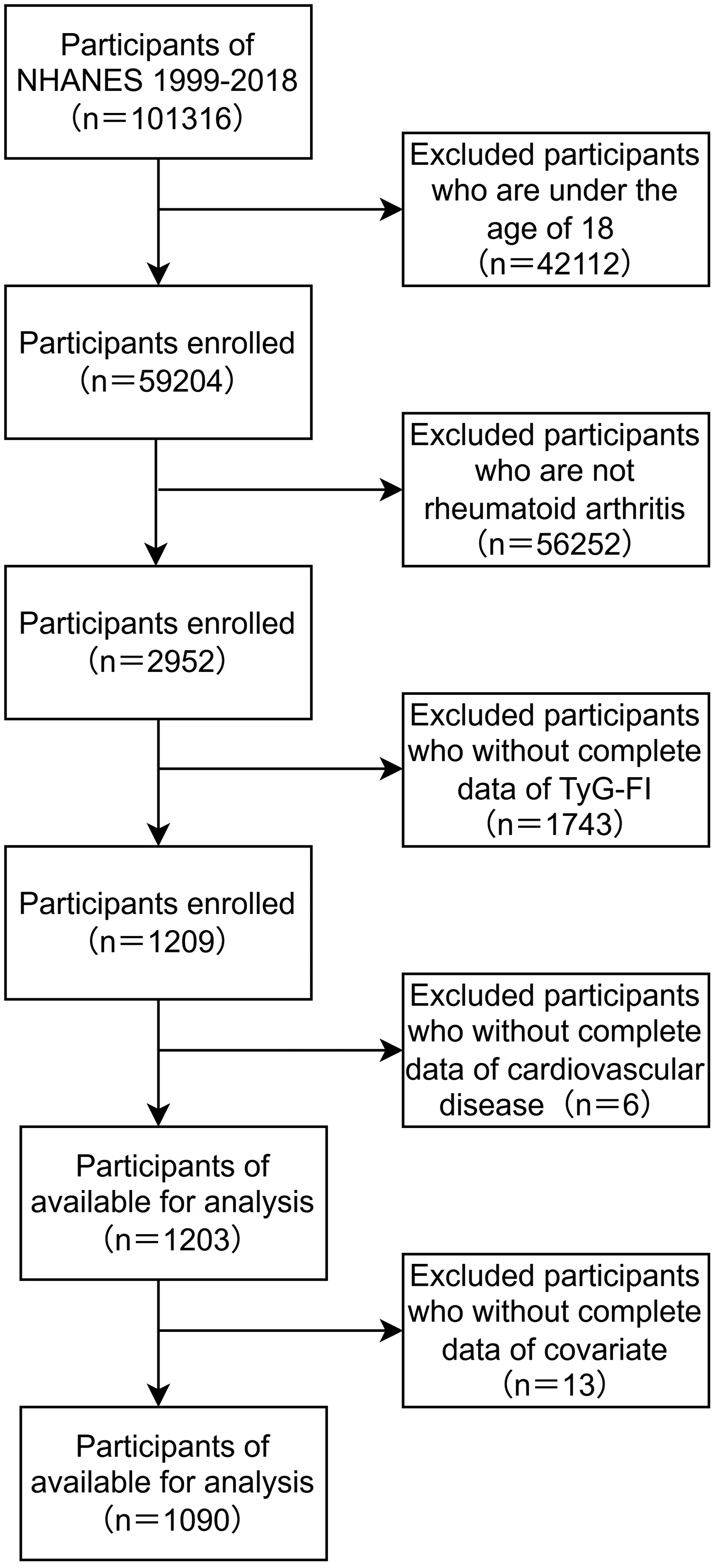
Researcher inclusion and exclusion flow chart. NHANES = National Health and Nutrition Examination Survey, TyG-FI = triglyceride-glucose–frailty index.

### 2.3. Definition of TyG-FI

The FI was constructed using 49 health deficits available in NHANES. These included 1 cognitive item; 15 items assessing activities and physical functioning; 7 depressive-symptom items; 13 chronic conditions; 5 measures of self-rated health, healthcare use, and medication burden; and 8 anthropometric, physical-performance, and laboratory measures. The complete list of items and their scoring criteria is provided in Table S5, Supplemental Digital Content 1.

Binary deficits were scored as 0 or 1. Variables with graded severity were assigned intermediate scores of 0.33, 0.50, or 0.66, as appropriate. For each participant, the FI was calculated as the sum of the individual deficit scores divided by 49. FI values ranged from 0 to 1, with higher values indicating greater deficit accumulation. The construction method of the FI index has been systematically validated in previous studies^[17,18]^:


$$\begin{array}{cc} & TyG\;index\\ & =ln\left[fasting\;triglycerides\left(mg/dL\right)\times fasting\;glucose\left(mg/dL\right)\right]/2. \end{array}$$

No cutoff was used to classify participants as frail or nonfrail. FI was treated as a continuous measure and multiplied by the TyG index to calculate TyG-FI.^[17]^ TyG-FI was analyzed continuously and by tertiles.

### 2.4. Definition of CVD

CVD data were determined through self-reported diagnoses obtained by doctors during individual interviews using standardized medical condition questionnaires. Participants were asked, “Has a doctor or other health professional told you that you have congestive heart failure/congenital heart disease/angina/myocardial infarction/stroke?” If “yes” was answered to any of the above questions, it was considered that the participant had CVD.

### 2.5. Source of mortality data

Data on mortality status were determined through linkage with the NHANES Public Use Linked Mortality File and the National Death Index. Follow-up time ranged from the initial interview date to the date of death, loss to follow-up, or the date of research review, with data cutoff on December 31, 2019.^[19]^

### 2.6. Covariates

Based on previous research, the covariates included in this study are age, gender, race, education level, marital status, smoking status, alcohol consumption status, body mass index (BMI), hypertension, diabetes, and estimated glomerular filtration rate (eGFR). These were collected through baseline questionnaires managed by trained professionals or measured in the NHANES laboratory. Participants provided information on age, gender (male or female), and race (Mexican American, other Hispanic, non-Hispanic White, non-Hispanic Black, other race). Age was categorized as ≤65 years and >65 years. Race was categorized as Mexican American, non-Hispanic White, non-Hispanic Black, and other race. Marital status was categorized as married or living with a partner, single (widowed/divorced/separated), and never married. Education level was categorized as less than high school, high school or equivalent, and college or higher. Smoking status was categorized as current smoker, former smoker, or never smoked, while alcohol consumption status was categorized as yes or no. BMI (weight [kg]/height [m^2^]) was calculated and categorized as <18.5, [18.5, 25), [25, 30), and≥30. Hypertension was diagnosed based on a history of hypertension, with systolic blood pressure ≥140 mm Hg and diastolic blood pressure ≥90 mm Hg. Diabetes was defined as a history of diabetes, HbA1c ≥6.5%, fasting glucose ≥126 mg/dL, or 2-hour postprandial glucose ≥200 mg/dL, or use of diabetes medication or insulin.

### 2.7. Statistical analysis

Based on the participants’ TyG-FI index levels, TyG-FI was used as a continuous variable and a categorical variable (divided into 3 groups based on tertiles: T1 [TyG-FI ≤ 0.82], T2 [0.82 < TyG-FI ≤ 1.42], and T3 [TyG-FI > 1.42], where the T1 group served as the reference group) to evaluate the relationship between TyG-FI and CVD and mortality in patients with RA. We used logistic regression, Cox proportional hazards models, Kaplan–Meier survival curves, threshold effect analysis, subgroup analysis, and unrestricted spline curves to determine the odds ratio (OR) or hazard ratio (HR) and 95% confidence interval (95% CI) of the TyG-FI index in relation to CVD and mortality in patients with RA. Model 1 was the crude model without adjustment for any potential confounding factors. Model 2 was adjusted for age and gender. Model 3 further adjusted for race, marital status, and education level. Model 4 included additional adjustments for BMI, smoking status, alcohol consumption, hypertension, diabetes, and eGFR. To explore the potential nonlinear dose–response relationship between the TyG-FI index and CVD and mortality in patients with RA, we employed a restricted cubic spline (RCS) model and adjusted for variables in Model 4. The TyG-FI index as a continuous variable was set with 4 knots at the 5th, 35th, 65th, and 95th percentiles. Based on the smooth curve, we used a 2-segment linear regression and logistic regression model to identify any threshold effects and adjusted for potential confounding factors. Exploratory subgroup analyses were conducted according to age, sex, smoking status, alcohol consumption, hypertension, and diabetes. Effect modification was evaluated using interaction terms between TyG-FI and the corresponding subgroup variables. Race-stratified outcome analyses were not performed because limited participant and event counts in some racial categories were expected to produce imprecise estimates. In addition, the area under the receiver operating characteristic (ROC) curve (AUC) was used to evaluate the ability of TyG-FI to discriminate CVD in patients with RA.

To evaluate the association between TyG-FI and CVD in patients with RA and their mortality, we conducted some sensitivity analyses: additional analyses were performed after imputing missing information on covariates. The results are shown in Table S1, Supplemental Digital Content 2; Table S2, Supplemental Digital Content 3; Table S3, Supplemental Digital Content 4; Table S6, Supplemental Digital Content 5; and Table S7, Supplemental Digital Content 6.

All analyses were performed using R 4.4.2 (http://www.R-project.org, R Foundation), and *P* values ≤.05 were considered statistically significant.

## 3. Results

As shown in Table 1, we enrolled 1090 participants, divided into 3 groups based on TyG-FI tertiles (T1: 321, T2: 370, T3: 399). An increasing TyG-FI index was associated with a progressive increase in cardiovascular risk factors and disease burden in RA patients. Specifically, the T3 group had older age (39.79% > 65 years), a higher proportion of females (66.00%), a lower education level (37.13% with high school or less education), and a significantly higher prevalence of obesity (61.69% with BMI ≥ 30), as well as significantly higher rates of hypertension, diabetes, and CVD compared with the T1 group (80.10% vs 34.99%, 49.66% vs 7.76%, 45.66% vs 3.62%, respectively). Cardiovascular mortality also showed an increasing trend (10.96% in the T3 group, 2.69% in the T1 group). Mean eGFR values decreased with increasing TyG-FI. This suggests that RA patients with a higher TyG-FI index exhibit a more prominent clustering of cardiovascular metabolic risk factors and poorer cardiovascular outcomes. The distribution of participants across TyG-FI tertiles differed by age and sex (*P* = .008 and *P* < .001, respectively), whereas no difference was observed across racial categories (*P* = .337).

**Table 1:** Population characteristics by TyG-FI index.

Variable	Total (n = 1090)	T1 (n = 321)	T2 (n = 370)	T3 (n = 399)	Statistic	*P*
Age, n (%)					χ^2^ = 17.37	.008
≤65	654 (68.09)	206 (74.45)	225 (69.61)	223 (60.21)		
>65	436 (31.91)	115 (25.55)	145 (30.39)	176 (39.79)		
Gender, n (%)					χ^2^ = 31.12	<.001
Male	482 (45.10)	166 (54.48)	173 (46.82)	143 (34.00)		
Female	608 (54.90)	155 (45.52)	197 (53.18)	256 (66.00)		
Race, n (%)					χ^2^ = 8.54	.337
Mexican American	175 (6.05)	54 (5.88)	59 (5.33)	62 (6.96)		
Non-Hispanic White	485 (69.33)	151 (70.94)	166 (71.05)	168 (65.97)		
Non-Hispanic Black	304 (15.04)	82 (11.77)	102 (15.16)	120 (18.18)		
Other Race	126 (9.57)	34 (11.41)	43 (8.47)	49 (8.88)		
Education, n (%)					χ^2^ = 40.46	<.001
Less than high school	393 (26.31)	91 (17.95)	123 (23.86)	179 (37.13)		
High school or equivalent	265 (28.15)	81 (27.52)	95 (31.48)	89 (25.34)		
College or above	432 (45.55)	149 (54.53)	152 (44.66)	131 (37.52)		
Marriage, n (%)					χ^2^ = 15.87	.046
Married or living with a partner	640 (67.20)	212 (72.89)	220 (69.15)	208 (59.55)		
Single (widowed/divorced/separated)	373 (26.90)	91 (21.88)	126 (25.07)	156 (33.76)		
Never married	77 (5.90)	18 (5.23)	24 (5.78)	35 (6.69)		
Smoking, n (%)					χ^2^ = 6.19	.506
Never	453 (36.39)	135 (37.51)	144 (34.59)	174 (37.12)		
Former	371 (35.75)	106 (35.15)	124 (33.39)	141 (38.77)		
Now	266 (27.86)	80 (27.35)	102 (32.01)	84 (24.11)		
Drinking alcohol, n (%)					χ^2^ = 19.45	.003
No	419 (35.58)	101 (27.90)	145 (35.19)	173 (43.63)		
Yes	671 (64.42)	220 (72.10)	225 (64.81)	226 (56.37)		
BMI, n (%)					χ^2^ = 113.54	<.001
<18.5	16 (1.55)	8 (2.31)	7 (2.23)	1 (0.10)		
[18.5, 25)	224 (22.82)	112 (36.52)	70 (19.52)	42 (12.61)		
[25, 30)	357 (31.72)	120 (35.16)	134 (34.34)	103 (25.60)		
≥30	493 (43.91)	81 (26.02)	159 (43.91)	253 (61.69)		
Hypertension, n (%)					χ^2^ = 149.72	<.001
No	385 (42.44)	177 (65.01)	129 (42.55)	79 (19.90)		
Yes	705 (57.56)	144 (34.99)	241 (57.45)	320 (80.10)		
Diabetes, n (%)					χ^2^ = 181.24	<.001
No	732 (74.77)	286 (92.24)	268 (81.62)	178 (50.34)		
Yes	358 (25.23)	35 (7.76)	102 (18.38)	221 (49.66)		
Cardiovascular disease, n (%)					χ^2^ = 169.42	<.001
No	800 (74.98)	305 (96.38)	270 (74.36)	225 (54.34)		
Yes	290 (25.02)	16 (3.62)	100 (25.64)	174 (45.66)		
Cardiovascular disease mortality, n (%)					χ^2^ = 19.75	<.001
No	994 (93.31)	311 (97.31)	330 (93.59)	353 (89.04)		
Yes	96 (6.69)	10 (2.69)	40 (6.41)	46 (10.96)		
eGFR, mean (SE)	87.12 (0.84)	90.16 (1.39)	89.04 (1.40)	82.12 (1.42)	*F* = 9.11	<.001
TyG, M (Q_1_, Q_3_)	8.76 (8.35, 9.19)	8.52 (8.15, 8.90)	8.73 (8.38, 9.07)	9.12 (8.57, 9.48)	χ^2^ = 73.40	<.001
FI, M (Q_1_, Q_3_)	0.20 (0.13, 0.29)	0.12 (0.09, 0.13)	0.20 (0.18, 0.24)	0.33 (0.28, 0.39)	χ^2^ = 2891.94	<.001
TyG-FI, M (Q_1_, Q_3_)	1.09 (0.68, 1.61)	0.55 (0.43, 0.68)	1.08 (0.96, 1.26)	1.85 (1.61, 2.41)	χ^2^ = 5296.12	<.001

χ^2^ = chi-square test, BMI = body mass index, eGFR = estimated glomerular filtration rate, *F* = analysis of variance, FI = frailty index, M = median, Q_1_ = first quartile, Q_3_ = third quartile, SE = standard error, TyG = triglyceride-glucose index, TyG-FI = triglyceride-glucose–frailty index.

### 3.1. The relationship between TyG-FI and CVD and its mortality

We evaluated the association between TyG-FI and CVD in patients with RA by constructing 4 multivariate logistic regression models, with the results shown in Table 2: elevated TyG-FI was significantly and independently associated with an increased risk of CVD in patients with RA. In all models, an increase of 1 unit in TyG-FI was associated with an increased risk of CVD (*P* < .05). After adjusting for age, gender, race, education level, marital status, smoking status, alcohol consumption, BMI, hypertension, diabetes, and eGFR in model 4, an increase of 1 unit in TyG-FI was significantly associated with an increased risk of CVD (OR = 4.50, 95% CI: 3.20–6.33). Compared with group T1, group T3 showed a significantly increased risk of CVD (OR = 19.46, 95% CI: 10.19–37.18). The results remained consistent across all models, demonstrating their robustness. These results indicate that TyG-FI, as a comprehensive indicator reflecting metabolic dysfunction and physiological vulnerability, has a strong and independent correlation with the risk of CVD in patients with RA, suggesting its potential value in cardiovascular risk stratification beyond traditional risk factors.

**Table 2:** The association between the TyG-FI index and cardiovascular disease.

Variables	Model 1		Model 2		Model 3		Model 4	
	OR (95% CI)	*P*	OR (95% CI)	*P*	OR (95% CI)	*P*	OR (95% CI)	*P*
TyG-FI	4.11 (3.11–5.42)	<.001	4.33 (3.22–5.83)	<.001	4.23 (3.14–5.70)	<.001	4.50 (3.20–6.33)	<.001
TyG-FI tertiles								
T1	1.00 (Reference)		1.00 (Reference)		1.00 (Reference)		1.00 (Reference)	
T2	9.19 (4.97–16.96)	<.001	9.40 (5.13–17.22)	<.001	9.21 (5.02–16.91)	<.001	9.00 (4.75–17.06)	<.001
T3	22.38 (12.60–39.76)	<.001	23.19 (12.90–41.70)	<.001	21.52 (12.04–38.48)	<.001	19.46 (10.19–37.18)	<.001

Model 1: crude.

Model 2: adjust: adjust for the following confounding factors: age, gender.

Model 3: adjust: adjust for the following confounding factors: age, gender, race, education level, marital status.

Model 4: adjust: adjust for the following confounding factors: age, gender, race, education level, marital status, smoking status, alcohol consumption, BMI, hypertension, diabetes, eGFR.

BMI = body mass index, CI = confidence interval, eGFR = estimated glomerular filtration rate, OR = odds ratio, TyG-FI = triglyceride-glucose–frailty index.

We evaluated the association between TyG-FI and CVD mortality in patients with RA by constructing a Cox proportional hazards regression model, with the results shown in Table 3: A significant and independent association was found between elevated TyG-FI and increased risk of CVD mortality in patients with RA. In all models, an increase of 1 unit in TyG-FI was associated with an increased risk of CVD mortality (*P* < .05). After adjusting for age, gender, race, education level, marital status, smoking status, alcohol consumption, BMI, hypertension, diabetes, and eGFR in model 4, an increase of 1 unit in TyG-FI was significantly associated with an increased risk of CVD mortality (HR = 1.77, 95% CI: 1.16–2.70). Compared with group T1, group T3 showed a significantly increased risk of CVD (HR = 4.72, 95% CI:1.60–13.89). The results remained consistent across all models, demonstrating their robustness. The findings further support the importance of TyG-FI as a comprehensive indicator reflecting metabolic dysfunction and physiological vulnerability in cardiovascular risk assessment for RA patients, suggesting it can serve as an effective biomarker for identifying patients at extremely high risk of cardiovascular death.

**Table 3:** Association between TyG-FI index and mortality from cardiovascular disease.

Variables	Model 1		Model 2		Model 3		Model 4	
	HR (95%CI)	*P*	HR (95%CI)	*P*	HR (95%CI)	*P*	HR (95%CI)	*P*
TyG-FI	2.21 (1.70–2.89)	<.001	2.14 (1.60–2.88)	<.001	2.05 (1.51–2.79)	<.001	1.77 (1.16–2.70)	.008
TyG-FI tertiles								
T1	1.00 (Reference)		1.00 (Reference)		1.00 (Reference)		1.00 (Reference)	
T2	2.43 (1.08–5.49)	.032	2.66 (1.29–5.50)	.008	2.91 (1.35–6.29)	.007	3.14 (1.23–8.00)	.017
T3	5.05 (2.12–12.00)	<.001	5.49 (2.43–12.42)	<.001	5.82 (2.60–13.02)	<.001	4.72 (1.60–13.89)	.005

Model 1: crude.

Model 2: adjust: adjust for the following confounding factors: age, gender.

Model 3: adjust: adjust for the following confounding factors: age, gender, race, education level, marital status.

Model 4: adjust: adjust for the following confounding factors: age, gender, race, education level, marital status, smoking status, alcohol consumption, BMI, hypertension, diabetes, eGFR.

BMI = body mass index, CI = confidence interval, eGFR = estimated glomerular filtration rate, HR = hazard ratio, TyG-FI = triglyceride-glucose–frailty index.

Furthermore, as shown in Table S4, Supplemental Digital Content 7, considering noncardiovascular death as a competing event, we conducted a Fine-Gray competing risk analysis, the results of which were largely consistent with the Cox model (subdistribution hazard ratio = 3.79, 95% CI: 1.01–14.23), supporting the robustness of the main conclusions.

### 3.2. RCS analysis and Kaplan–Meier survival curves exploring the relationship between TyG-FI and CVD and mortality outcomes

The analysis of the restricted spline curve is shown in Figure 2, demonstrating a significant nonlinear positive correlation between the TyG-FI index and CVD, as well as its mortality. For the association between TyG-FI and CVD (panel A), the correlation is overall highly significant (*P* for overall < .001) and shows a clear nonlinear pattern (*P* for nonlinear < .001). The curve reveals a significant “J-shaped” relationship. The reference value for the curve is 0.924, with CVD risk gradually increasing at lower TyG-FI levels (TyG-FI < 0.924) and sharply accelerating after TyG-FI >0.924, meaning that as TyG-FI values increase, the risk of CVD rises sharply. For the association between TyG-FI and CVD mortality (panel B), the correlation is overall highly significant (*P* for overall = .002) and shows a clear nonlinear pattern (*P* for nonlinear = .012). The reference value for the curve is 1.079, with CVD mortality risk gradually increasing at lower TyG-FI levels (TyG-FI < 1.079) and rising slowly after TyG-FI > 1.079. These findings confirm that TyG-FI is a nonlinear predictor of adverse cardiovascular outcomes in RA patients. The significant rise in CVD risk above the threshold highlights the potential of TyG-FI in identifying high-risk subgroups, while the association between TyG-FI and CVD mortality emphasizes its prognostic value within the range of CVD severity in this population.

**Figure 2.**
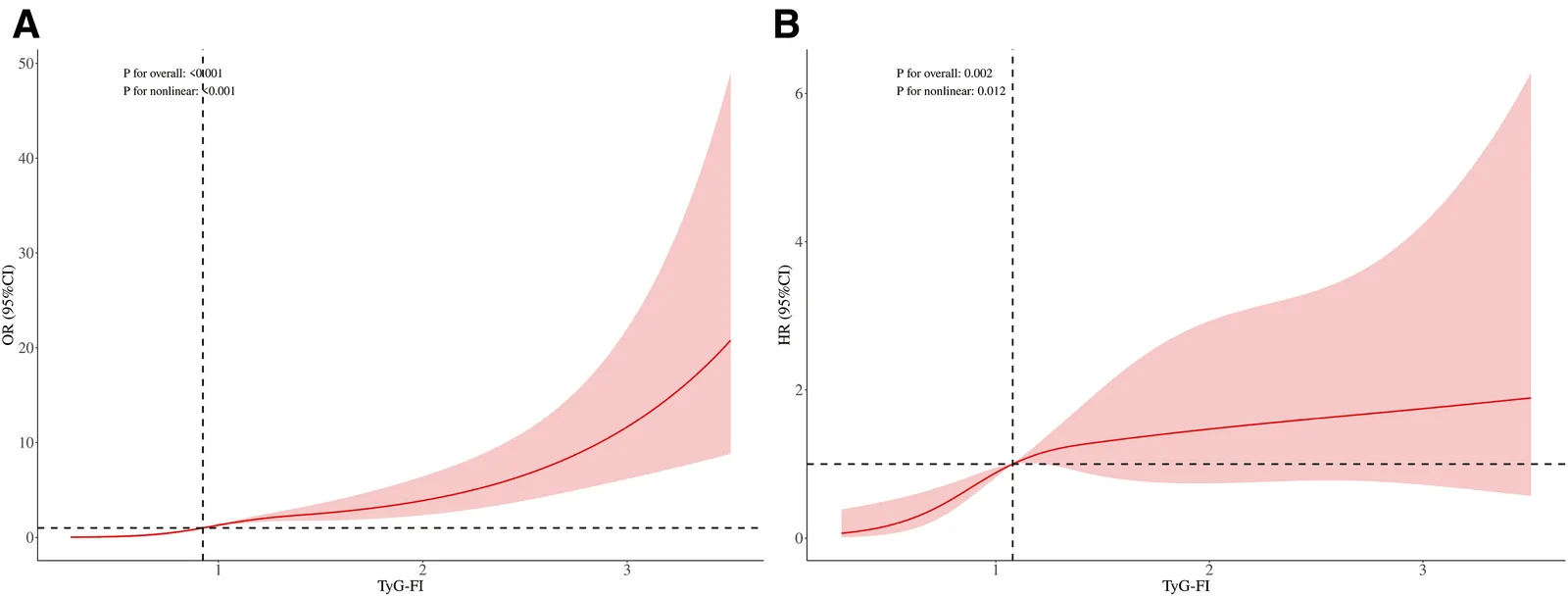
Nonrestricted cubic spline models of the relationship between the TyG-FI index and cardiovascular disease (A) and cardiovascular disease mortality (B), adjusted for age, gender, race, education level, marital status, smoking status, alcohol consumption, BMI, hypertension, diabetes, and eGFR. BMI = body mass index, CI = confidence interval, eGFR = estimated glomerular filtration rate, OR = odds ratio, TyG-FI = triglyceride-glucose–frailty index.

However, in areas with higher TyG-FI values, the confidence interval widens significantly, suggesting that the data points within this interval are relatively sparse, leading to a decrease in the precision of the estimated values. Therefore, interpretation of specific risk values under high TyG-FI levels should be approached with caution, although the overall upward trend is clear.

We performed a Kaplan–Meier analysis on the CVD mortality in patients with RA based on TyG-FI. The results, as shown in Figure 3, indicate a log-rank *P* < .001, strongly statistically demonstrating fundamental differences in survival time distribution among the 3 groups, with TyG-FI showing a positive correlation with CVD mortality in patients with RA. The T3 group with the highest TyG-FI exhibited the steepest decline in survival rate, indicating the highest risk of cardiovascular death; the T2 group showed an intermediate risk; and the T1 group had the highest survival rate and the best prognosis. This “T3 > T2 > T1” risk gradient is consistent with the previously observed large HR in the Cox regression, confirming that TyG-FI is a powerful and independent stratifying factor for cardiovascular death risk in patients with RA.

**Figure 3.**
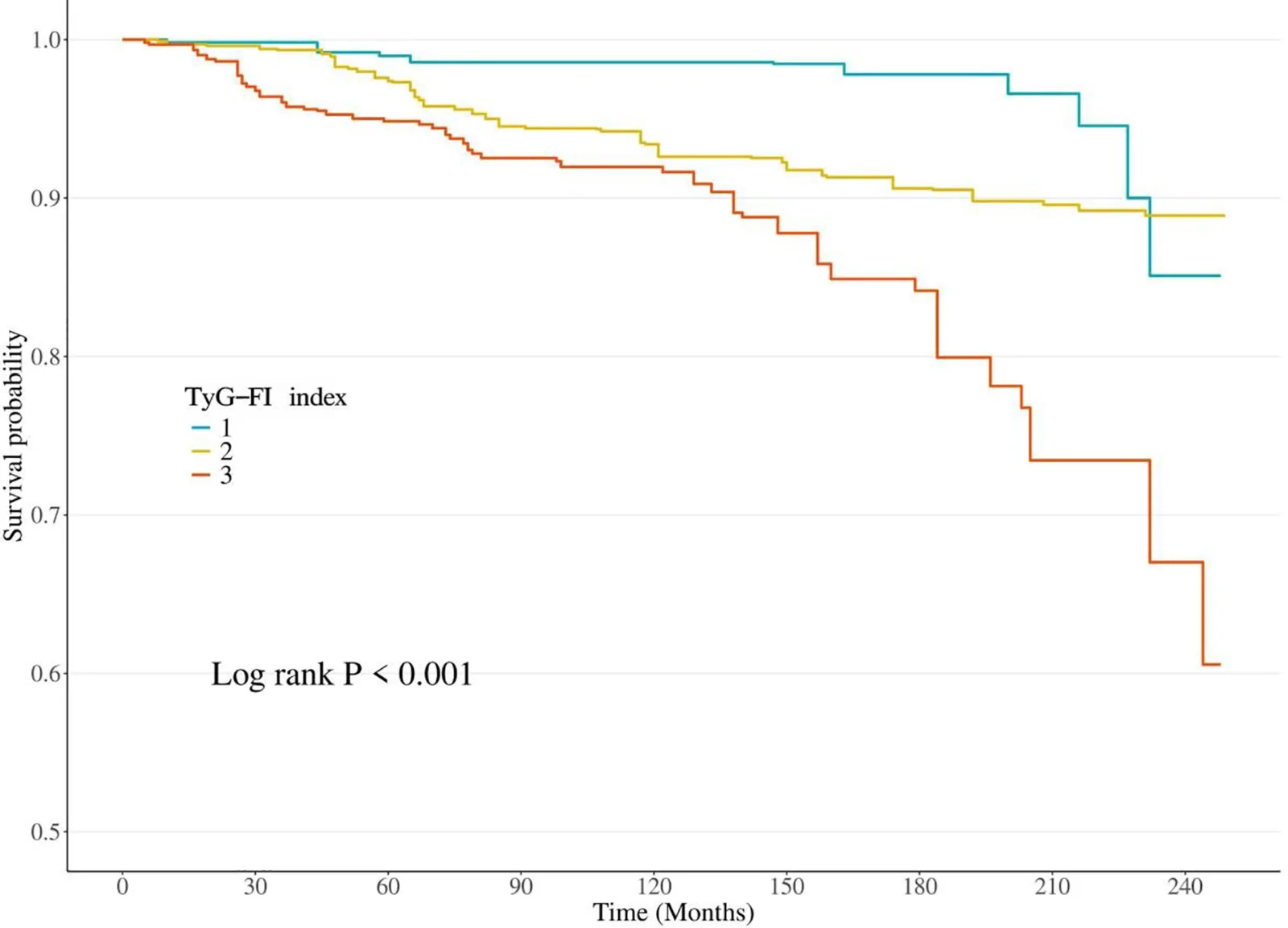
Kaplan–Meier analysis of cardiovascular disease mortality in patients with rheumatoid arthritis based on TyG-FI. TyG-FI = triglyceride-glucose–frailty index.

### 3.3. A comparative analysis of the discriminative value of TyG and TyG-FI for CVDs and mortality outcomes

ROC analysis was conducted to evaluate the ability of TyG-FI and TyG to discriminate CVD and its risk of mortality. The results are shown in Figure 4: The TyG-FI index showed good discriminative ability in discriminating CVD and its risk of mortality in patients with RA. The AUC (combined demographic, lifestyle, and clinical variables) for TyG-FI discriminating CVD and its risk of mortality were 0.80 and 0.81, respectively. The AUC (combined demographic, lifestyle, and clinical variables) for TyG discriminating CVD and its risk of mortality were 0.73 and 0.80, respectively. Compared with the TyG index, the TyG-FI index demonstrated better discriminative ability in discriminating CVD and its risk of mortality in patients with RA, highlighting the superiority of TyG-FI over the single TyG in discriminating CVD and mortality.

**Figure 4.**
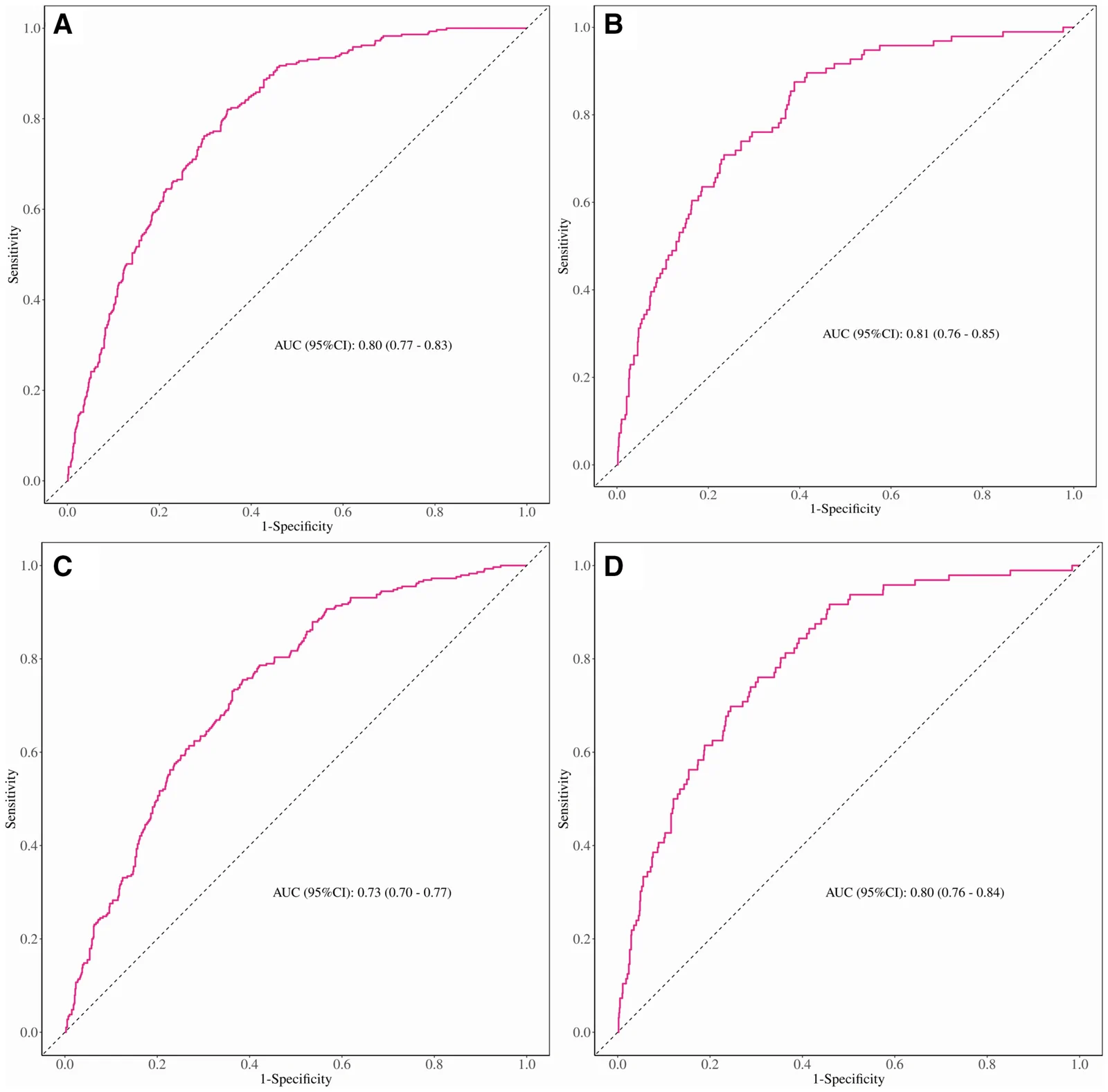
Receiver operating characteristic curves for the TyG-FI index in discriminating cardiovascular disease (A) and cardiovascular disease mortality (B) in adult American rheumatoid arthritis patients, and for the TyG index in discriminating cardiovascular disease (C) and cardiovascular disease mortality (D) in adult American rheumatoid arthritis patients, adjusted for age, gender, race, education level, marital status, smoking status, alcohol consumption, BMI, hypertension, diabetes, and eGFR. AUC = area under the receiver operating characteristic curve, BMI = body mass index, CI = confidence interval, eGFR = estimated glomerular filtration rate, TyG-FI = triglyceride-glucose–frailty index.

### 3.4. TyG-FI index and subgroup analysis of CVD and its mortality rates

The subgroup analysis results of the TyG-FI index and CVD and its mortality rates are shown in Figure 5: An elevated TyG-FI index was positively correlated with a higher CVD risk (OR > 1) and its mortality (HR > 1) (*P* values all < .05). In the association with CVD, age was suggested to be a potential effect modifier (*P* for interaction = .044). In patients aged ≤65, the association between TyG-FI and CVD was significantly stronger (OR = 7.07) compared with older patients aged >65 (OR = 2.81). This indicates that in younger RA patients, elevated TyG-FI is an especially strong independent risk signal, with potentially greater discriminative value. This result is consistent with previous studies: CTI demonstrates stronger predictive efficacy for CVD mortality in younger individuals.^[19]^ The association between TyG-FI and CVD is significantly stronger in RA patients aged ≤65 years than in elderly patients.^[20]^ In elderly RA patients, traditional age-related CVD risk factors (such as vascular aging, long-term hypertension) dominate, thereby partially masking the additional effect of RA-specific inflammatory-driven metabolic disorders and debility on CVD risk. In relatively younger RA patients, the increased CVD risk is primarily attributed to chronic inflammation, IR, and the decline in physiological reserves caused by the disease itself.^[21]^ As a composite indicator integrating metabolic disorders (TyG) and overall health status (FI), TyG-FI can more sensitively capture this additional cardiovascular risk driven by the “RA-specific burden” in younger RA patients. This finding highlights the importance of the TyG-FI index in early identification and intervention of metabolic and debility status in younger RA patients. In the association with CVD mortality, the TyG-FI index was positively correlated with CVD mortality risk, with no significant interaction observed between TyG-FI and subgroup variables.

**Figure 5.**
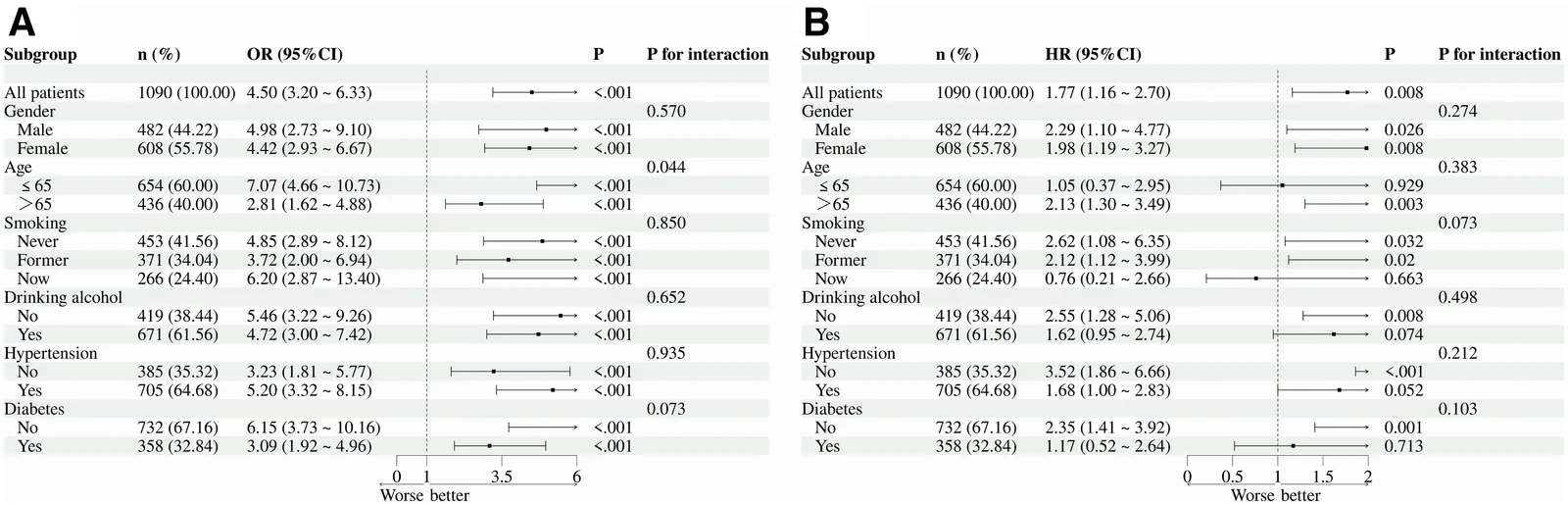
Forest plot of subgroup analysis of cardiovascular disease (A) and its mortality (B) in rheumatoid arthritis patients with TyG-FI, adjusted for age, gender, race, education level, marital status, smoking status, alcohol consumption, BMI, hypertension, diabetes, and eGFR. BMI = body mass index, CI = confidence interval, eGFR = estimated glomerular filtration rate, HR = hazard ratio, OR = odds ratio, TyG-FI = triglyceride-glucose–frailty index.

### 3.5. Sensitivity analysis

To enhance the robustness of the results, missing data imputation was performed using linear and logistic regression models, with final imputed values derived through nearest neighbor matching based on the predictive mean matching framework. We conducted additional analyses after imputing missing data. The results are shown in Table S2, Supplemental Digital Content 3 and Table S3, Supplemental Digital Content 4: TyG-FI is positively correlated with CVD and mortality in patients with RA, consistent with previous research findings, indicating a very strong association, with minimal influence from potential confounding factors or missing data processing.

## 4. Discussion

This study, based on the NHANES database with national representativeness, for the first time demonstrates a significant association between TyG-FI and CVD and mortality in American adult RA patients. After adjusting for demographic, lifestyle, and clinical factors, higher TyG-FI remained significantly positively correlated with CVD and its mortality risk. Smooth curve fitting showed a significant nonlinear relationship between the TyG-FI index and CVD (*P* for nonlinear < .001) and CVD mortality (*P* for nonlinear = .012). Kaplan–Meier analysis strongly supported the significant association between TyG-FI and CVD mortality. ROC analyses yielded AUCs of 0.73 and 0.80 for models incorporating TyG and 0.80 and 0.81 for models incorporating TyG-FI for prevalent CVD and cardiovascular mortality, respectively. These within-sample estimates should be interpreted cautiously. AUC summarizes ranking discrimination across possible thresholds but does not evaluate calibration, absolute risk, performance at a clinically relevant threshold, or the clinical consequences of false-positive and false-negative classifications. Although the conventional ROC AUC is not directly determined by outcome prevalence, positive and negative predictive values are prevalence-dependent, and differences in case mix may affect the observed discrimination. The numerical differences in AUC therefore do not establish the clinical superiority or clinical utility of TyG-FI. Subgroup and sensitivity analyses provided additional exploratory evidence regarding the consistency of the observed associations. These results suggest that TyG-FI, as an easily obtainable integrated biomarker, is highly likely to reflect the comprehensive impact of metabolic dysfunction and frailty on physiological health status.

TyG-FI integrates indicators of metabolic function and physiological health status from multiple dimensions, demonstrating excellent discriminative ability. As a reliable alternative index for IR,^[22–24]^ the elevation of the TyG index directly reflects widespread metabolic dysregulation in RA patients. Chronic inflammation is a core feature of RA,^[25]^ not only driving joint destruction but also being a key link in the occurrence of IR and dyslipidemia.^[26]^ Inflammatory factors such as tumor necrosis factor-α can directly interfere with the insulin signaling pathway, promoting IR,^[27]^ while IR further exacerbates endothelial dysfunction, promotes oxidative stress, and accelerates atherosclerotic plaque formation.^[28]^ This study confirms that high TyG-FI is associated with higher prevalence rates of diabetes and obesity, which is fully consistent with the aforementioned “inflammation-metabolism” theory.

The physiological decline in RA patients is often overlooked, but the introduction of the FI addresses this deficiency.^[14]^ Frailty occurs at a very high rate in RA patients, significantly higher than in age-matched healthy populations.^[16,29–31]^ It is not simply a result of aging but rather a state of reduced physiological reserve and multisystem dysfunction caused by chronic inflammation, hormonal disorders, malnutrition, sarcopenia, and multimorbidity.^[32]^ In addition, systemic inflammation may increase the risk of frailty and metabolic syndrome in RA patients.^[33]^ In this study, the high TyG-FI group exhibited lower eGFR, higher age, and greater comorbidity burden, reflecting this systemic dysfunction. Frailty indicates severe impairment in the body’s ability to cope with physiological stress, directly leading to a significant increase in the risk of adverse outcomes.^[34]^

This study supports and deepens the understanding of the association between IR and cardiovascular risk in RA.^[35,36]^ Previous research has confirmed that the TyG index is significantly associated with disease activity and subclinical atherosclerosis in RA patients.^[37,38]^ We advanced this association’s endpoint from intermediate markers to the diagnosis of CVD and its mortality, confirming that the IR status represented by the TyG index is a direct driving factor of adverse cardiovascular outcomes in RA patients.^[39,40]^ General-population cardiovascular risk models, including the Framingham Risk Score, may not perform consistently in patients with RA because they primarily incorporate conventional risk factors and may not fully capture RA-related metabolic and physiological burden.^[6,7]^ The rationale for examining TyG-FI lies in the complementary information represented by its components, rather than an assumption that it is superior to established risk models. TyG reflects IR and metabolic dysfunction,^[11]^ whereas FI summarizes accumulated deficits across functional, psychological, clinical, and physiological domains.^[14]^ Their combination may therefore characterize the joint metabolic and multidimensional health burden associated with RA. Previous population-based evidence linking TyG-FI with cardiovascular outcomes provided an additional basis for examining this composite index.^[17]^ From a practical perspective, TyG-FI may summarize 2 clinically relevant domains: metabolic dysfunction and accumulated health deficits.^[11]^ A higher TyG-FI value could prompt a more comprehensive cardiovascular assessment alongside established risk tools. The present analyses were conducted within a single NHANES sample and did not evaluate calibration, incremental value beyond established cardiovascular risk models, clinical net benefit, or transportability to other populations.^[41,42]^ External validation in independent prospective RA cohorts is therefore required before clinical thresholds or routine use can be proposed. Future studies should compare TyG-FI with established cardiovascular risk markers and models, assess calibration and discrimination at a prespecified time horizon, and determine whether adding TyG-FI improves clinical decision-making.^[43]^ RA care increasingly incorporates targeted therapies and individualized management strategies.^[44]^ Prospective validation studies should therefore collect detailed information on disease characteristics and longitudinal treatment exposure when examining heterogeneity in TyG-FI associations. Data-rich approaches, including artificial intelligence-assisted predictive modeling, may help integrate clinical, laboratory, imaging, genetic, and lifestyle information, although such applications require dedicated development and external validation in RA populations.^[45]^

This study has several limitations. Its observational design precludes causal inference, and the cross-sectional assessment of prevalent CVD does not establish whether TyG-FI preceded the development of CVD.^[46]^ The multivariable estimates remain susceptible to residual confounding, multicollinearity, and model misspecification.^[47,48]^ Because prevalent CVD was not rare, the odds ratios should not be interpreted as risk ratios.^[49]^ The nonlinear associations observed in the RCS analyses indicate that estimates based on a single linear TyG-FI term represent summary associations rather than constant associations across the observed range.^[50]^ Estimates from the Cox models depend on the proportional hazards and noninformative censoring assumptions, and potential violations cannot be excluded. The relatively limited number of cardiovascular deaths compared with the number of adjusted covariates may have reduced precision and increased the risk of overfitting. The handling of missing covariates also relies on assumptions about the missing-data mechanism.^[51]^ Sample-derived tertiles, threshold estimates, and subgroup findings were exploratory and should not be interpreted as validated clinical cutoffs. The AUC summarizes discrimination within the analytical sample but does not assess calibration, clinical utility, or performance in independent populations.^[52]^

Participant selection may also have affected the estimates. RA was identified from self-reported physician diagnosis rather than independent clinical verification, leaving the possibility of disease misclassification. Eligibility further depended on the availability of fasting laboratory measurements and complete information on TyG-FI components, covariates, and outcomes.^[53]^ Participants who completed these assessments may have differed from those with incomplete examinations in health status, healthcare access, or cardiovascular risk. The direction and magnitude of the resulting selection bias cannot be determined. NHANES surveys the noninstitutionalized US population; institutionalized individuals, who may have greater frailty, more severe RA, or higher cardiovascular risk, were not represented.^[54]^

RA treatment was not included in the current models. Glucocorticoids may worsen glucose and lipid metabolism and increase cardiovascular risk, whereas conventional synthetic disease-modifying antirheumatic drugs and biologic agents may reduce systemic inflammation and alter metabolic and cardiovascular risk.^[55]^ Treatment selection is also related to RA severity, creating potential confounding by indication. Experimental arthritis models have also been used to evaluate candidate anti-inflammatory and anti-arthritic therapies, although findings from such models cannot be extrapolated directly to cardiovascular outcomes in human RA.^[56]^ The NHANES prescription medication files capture medication use during the 30 days before the interview but do not provide consistently harmonized information across survey cycles on RA-specific indications, dosage, cumulative exposure, adherence, or treatment changes. We could not characterize long-term treatment regimens or determine their temporal relationship with TyG-FI and CVD. Residual confounding by disease severity and treatment exposure therefore remains possible.^[57]^

Prevalent CVD was primarily identified from self-reported physician diagnoses and may be affected by recall error or misclassification. Participants with unrecognized, asymptomatic, or atypical CVD may have been classified as not having CVD.^[58]^ Cardiovascular mortality was ascertained through linkage to the National Death Index, but errors in record linkage or cause-of-death classification remain possible.^[59]^

The FI was constructed from variables available in NHANES. Although it followed the deficit-accumulation approach, it may not be directly interchangeable with clinically administered or independently validated frailty instruments. A single baseline TyG-FI measurement could not capture changes in metabolic and frailty status during follow-up. The findings should be generalized cautiously beyond US noninstitutionalized adults with self-reported RA, particularly to institutionalized patients and populations with different racial, ethnic, healthcare, or treatment characteristics.^[60]^

## 5. Conclusion

Higher TyG-FI was associated with prevalent CVD and cardiovascular mortality among US adults with self-reported RA. The index may capture complementary information on metabolic dysfunction and accumulated health deficits. Although models incorporating TyG-FI showed greater numerical discrimination than those incorporating TyG alone, the incremental predictive value and clinical relevance of this difference remain to be established. Prospective studies are needed to determine clinically meaningful thresholds, evaluate calibration and clinical net benefit, compare TyG-FI with established cardiovascular risk models, and validate its performance in independent populations before routine clinical use.

## Acknowledgments

We sincerely appreciate the NHANES database for all the data.

## Author contributions

**Conceptualization:** Yangyu Xu, Hong Zhao, Liyun Zhang.

**Data curation:** Yangyu Xu, Hong Zhao, Jiannan Han, Liyun Zhang, Junkang Zhao.

**Formal analysis:** Yangyu Xu, Yuxiang Gao, Jiannan Han, Zewen Wu.

**Methodology:** Yangyu Xu, Yuxiang Gao, Jiannan Han, Zewen Wu, Junkang Zhao.

**Writing – original draft:** Yangyu Xu.

**Writing – review & editing:** Yangyu Xu.

**Investigation:** Hong Zhao, Jiannan Han.

**Resources:** Yuxiang Gao, Zewen Wu, Liyun Zhang, Junkang Zhao.

**Supervision:** Yuxiang Gao.

**Validation:** Yuxiang Gao.

**Project administration:** Zewen Wu.

**Visualization:** Zewen Wu.

**Funding acquisition:** Liyun Zhang, Junkang Zhao.

**Software:** Junkang Zhao.














