# Supplementary material for: Comparison analysis of the triglyceride-glucose index, triglyceride-glucose and frailty index in cardiovascular disease and mortality association among patients with rheumatoid arthritis: A population-based cohort study
Source: Medicine (Baltimore). 2026 Oct 16;105(42):e51143. doi: 10.1097/MD.0000000000051143 (PMC13681120; doi:10.1097/MD.0000000000051143)
Supplement: Supplementary file 1 [file medi-105-e51143-s001.docx]

**Table S5.** Variables in The 49-Item Frailty Index and Their Respective Scorings.

| Variable | Scoring | |
| --- | --- | --- |
| 1. Experience confusion/memory problems | Yes=1, No=0 | |
| 2. Managing money | Difficulty=1, No Difficulty=0 | |
| 3. Stooping, crouching, kneeling | Difficulty=1, No Difficulty=0 | |
| 4. Lifting or carrying | Difficulty=1, No Difficulty=0 | |
| 5. House chore | Difficulty=1, No Difficulty=0 | |
| 6. Preparing meals | Difficulty=1, No Difficulty=0 | |
| 7. Standing up from armless chair | Difficulty=1, No Difficulty=0 | |
| 8. Getting in and out of bed difficulty | Difficulty=1, No Difficulty=0 | |
| 9. Using fork, knife, drinking from cup | Difficulty=1, No Difficulty=0 | |
| 10. Dressing yourself | Difficulty=1, No Difficulty=0 | |
| 11. Standing for long periods difficulty | Difficulty=1, No Difficulty=0 | |
| 12. Grasp/holding small objects | Difficulty=1, No Difficulty=0 | |
| 13. Attending social event | Difficulty=1, No Difficulty=0 | |
| 14. Push or pull large objects | Difficulty=1, No Difficulty=0 | |
| 15. Walking for a quarter mile difficulty | Difficulty=1, No Difficulty=0 | |
| 16. Walking up ten steps difficulty | Difficulty=1, No Difficulty=0 | |
| 17. Have little interest in doing things | Nearly every day=1, More than half the days= 0.66, Several days= 0.33, Not at all=0 | |
| 18. Feeling down, depressed, or hopeless | Nearly every day=1, More than half the days= 0.66, Several days= 0.33, Not at all=0 | |
| 19. Trouble sleeping or sleeping too much | Nearly every day=1, More than half the days= 0.66, Several days= 0.33, Not at all=0 | |
| 20. Feeling tired or having little energy | Nearly every day=1, More than half the days= 0.66, Several days= 0.33, Not at all=0 | |
| 21. Poor appetite or overeating | Nearly every day=1, More than half the days= 0.66, Several days= 0.33, Not at all=0 | |
| 22. Feeling bad about yourself | Nearly every day=1, More than half the days= 0.66, Several days= 0.33, Not at all=0 | |
| 23. trouble concentrating on things | Nearly every day=1, More than half the days= 0.66, Several days= 0.33, Not at all=0 | |
| 24. Arthritis | Yes=1, Suspect = 0.5 No=0 | |
| 25. Thyroid problems | Yes=1, Suspect = 0.5 No=0 | |
| 26. Chronic Bronchitis | Yes=1, Suspect = 0.5 No=0 | |
| 27. Cancer | Yes=1, Suspect = 0.5 No=0 | |
| 28. Congestive Heart Failure | Yes=1, Suspect = 0.5 No=0 | |
| 29. Coronary Heart Disease | Yes=1, Suspect = 0.5 No=0 | |
| 30. Angina | Yes=1, Suspect = 0.5 No=0 | |
| 31. Heart Attack | Yes=1, Suspect = 0.5 No=0 | |
| 32. Stroke | Yes=1, Suspect = 0.5 No=0 | |
| 33. Blood Pressure | Yes=1, Suspect = 0.5 No=0 | |
| 34. Diabetes | Yes=1, Suspect = 0.5 No=0 | |
| 35. weak/failing kidneys | Yes=1, Suspect = 0.5 No=0 | |
| 36. Urinary Leakage | Yes=1, Suspect = 0.5 No=0 | |
| 37. Self Rated Health | Fair, poor=1, Excellent, Very good, good=0 | |
| 38. Health now compared with 1 year ago | Worse=1, About the same, better =0 | |
| 39. Overnight hospital patient in last year | Yes=1, No=0 | |
| 40. Frequency of health care use during last year. | None=0, 1-5= 0,5, More than 5=1 | |
| 41. Number of Prescribed medications | None=0 , 1-4=0.5, Five and more= 1 | |
| 42. Body mass index | <18.5, ≥ 30 =1  25-<30 = 0.5  18.5-25 =0 | |
| 43. Handgrip strength | MALES:  For BMI ≤ 24, GS ≤ 29  For BMI 24.1–28, GS ≤ 30  For BMI >28, GS ≤ 32 =1 | FEMALES:  For BMI ≤ 23, GS ≤ 17  For BMI 23.1–26, GS ≤ 17.3  For BMI 26.1–29, GS ≤ 18  For BMI>29, GS ≤ 21 =1 |
| 44. Glycohemoglobin (%) | 0-5.7 % =0, >5.7% =1 | |
| 45. Red blood cell count (million cells/μL) | M: 4.7-6.1= 0, Other =1 | F: 4.2-5.4=0, Other =1 |
| 46. Hemoglobin (g/dL) | M: 13.5-18= 0, Other=1 | F: 12-16 =0, Other = 1 |
| 47. Red cell distribution width (%) | 11.6-14.6 = 0, Other =1 | |
| 48. Lymphocyte percent (%) | 20- 40 = 0, Other=1 | |
| 49. Segmented neutrophils percent (%) | 40-80 = 0, Other=1 | |
