# Supplementary material for: Comparison analysis of the triglyceride-glucose index, triglyceride-glucose and frailty index in cardiovascular disease and mortality association among patients with rheumatoid arthritis: A population-based cohort study
Source: Medicine (Baltimore). 2026 Oct 16;105(42):e51143. doi: 10.1097/MD.0000000000051143 (PMC13681120; doi:10.1097/MD.0000000000051143)
Supplement: Supplementary file 2 [file medi-105-e51143-s002.docx]

**Table S1.** Population characteristics classified according to the TyG-FI index after imputing missing covariate data.

| Variable | | Total (n = 1209) | T1 (n=348) | T2 (n=419) | T3 (n=442) | Statistic | P |
| --- | --- | --- | --- | --- | --- | --- | --- |
| Age, n(%) | |  |  |  |  | χ²=17.18 | 0.010 |
| ≤65 | | 736 (68.74) | 229 (75.41) | 256 (69.03) | 251 (61.84) |  |  |
| ＞65 | | 473 (31.26) | 119 (24.59) | 163 (30.97) | 191 (38.16) |  |  |
| Gender, n(%) | |  |  |  |  | χ²=43.56 | <.001 |
| Male | | 527 (45.19) | 184 (56.42) | 193 (46.01) | 150 (33.24) |  |  |
| Female | | 682 (54.81) | 164 (43.58) | 226 (53.99) | 292 (66.76) |  |  |
| Race, n(%) | |  |  |  |  | χ²=10.44 | 0.213 |
| Mexican American | | 202 (6.39) | 58 (6.04) | 73 (6.24) | 71 (6.91) |  |  |
| Non-Hispanic White | | 514 (67.91) | 161 (70.68) | 175 (68.87) | 178 (64.20) |  |  |
| Non-Hispanic Black | | 344 (15.87) | 88 (11.90) | 120 (16.19) | 136 (19.47) |  |  |
| Other Race | | 149 (9.83) | 41 (11.39) | 51 (8.71) | 57 (9.42) |  |  |
| Education, n(%) | |  |  |  |  | χ²=37.97 | <.001 |
| Less than high school | | 443 (26.98) | 99 (19.38) | 147 (24.79) | 197 (36.70) |  |  |
| high school or equivalent | | 294 (28.17) | 89 (27.10) | 106 (32.04) | 99 (25.29) |  |  |
| college or above | | 472 (44.86) | 160 (53.51) | 166 (43.17) | 146 (38.01) |  |  |
| Marriage, n(%) | |  |  |  |  | χ²=19.53 | 0.022 |
| Married or living with a partner | | 693 (65.23) | 224 (70.77) | 247 (67.99) | 222 (56.96) |  |  |
| Single(widowed/divorced/separated) | | 424 (28.31) | 101 (23.04) | 147 (26.47) | 176 (35.38) |  |  |
| Never married | | 92 (6.46) | 23 (6.19) | 25 (5.55) | 44 (7.66) |  |  |
| Smoking, n(%) | |  |  |  |  | χ²=3.87 | 0.730 |
| Never | | 515 (36.67) | 153 (37.45) | 167 (35.68) | 195 (36.90) |  |  |
| Former | | 396 (35.17) | 108 (34.45) | 138 (33.27) | 150 (37.83) |  |  |
| Now | | 298 (28.16) | 87 (28.10) | 114 (31.05) | 97 (25.27) |  |  |
| Drinking alcohol, n(%) | |  |  |  |  | χ²=22.78 | 0.002 |
| No | | 469 (35.73) | 111 (27.43) | 165 (36.06) | 193 (43.59) |  |  |
| Yes | | 740 (64.27) | 237 (72.57) | 254 (63.94) | 249 (56.41) |  |  |
| BMI, n(%) | |  |  |  |  | χ²=123.41 | <.001 |
| <18.5 | | 17 (1.49) | 8 (2.13) | 8 (2.26) | 1 (0.09) |  |  |
| [18.5,25) | | 253 (23.16) | 123 (36.40) | 80 (19.39) | 50 (13.91) |  |  |
| [25,30) | | 391 (31.57) | 125 (35.49) | 155 (34.82) | 111 (24.38) |  |  |
| ≥30 | | 548 (43.78) | 92 (25.98) | 176 (43.53) | 280 (61.62) |  |  |
| Hypertension, n(%) | |  |  |  |  | χ²=167.51 | <.001 |
| No | | 427 (42.01) | 191 (64.85) | 148 (41.76) | 88 (19.68) |  |  |
| Yes | | 782 (57.99) | 157 (35.15) | 271 (58.24) | 354 (80.32) |  |  |
| Diabetes, n(%) | |  |  |  |  | χ²=196.87 | <.001 |
| No | | 810 (74.39) | 311 (92.56) | 301 (80.22) | 198 (50.49) |  |  |
| Yes | | 399 (25.61) | 37 (7.44) | 118 (19.78) | 244 (49.51) |  |  |
| Cardiovascular disease , n(%) | |  |  |  |  | χ²=186.54 | <.001 |
| No | | 898 (75.66) | 331 (96.58) | 312 (75.50) | 255 (55.13) |  |  |
| Yes | | 311 (24.34) | 17 (3.42) | 107 (24.50) | 187 (44.87) |  |  |
| Died of cardiovascular disease , n(%) | |  |  |  |  | χ²=25.56 | <.001 |
| No | | 1106 (93.38) | 338 (97.52) | 377 (93.96) | 391 (88.69) |  |  |
| Yes | | 103 (6.62) | 10 (2.48) | 42 (6.04) | 51 (11.31) |  |  |
| eGFR, Mean (SE) | | 87.32 (0.79) | 90.82 (1.29) | 88.68 (1.33) | 82.47 (1.34) | F=10.33 | <.001 |
|  | SE: Standard Error | | | | | | |
|  | F: ANOVA, χ²: Chi-square test | | | | | | |
