# Supplementary material for: Comparison analysis of the triglyceride-glucose index, triglyceride-glucose and frailty index in cardiovascular disease and mortality association among patients with rheumatoid arthritis: A population-based cohort study
Source: Medicine (Baltimore). 2026 Oct 16;105(42):e51143. doi: 10.1097/MD.0000000000051143 (PMC13681120; doi:10.1097/MD.0000000000051143)
Supplement: Supplementary file 3 [file medi-105-e51143-s003.docx]

**Table S2.** The association between the TyG-FI index and cardiovascular disease after processing the missing covariate data by imputation.

| Variables | Model1 | |  | Model2 | |  | Model3 | |  | Model4 | |
| --- | --- | --- | --- | --- | --- | --- | --- | --- | --- | --- | --- |
|  | OR (95%CI) | *P* |  | OR (95%CI) | *P* |  | OR (95%CI) | *P* |  | OR (95%CI) | *P* |
| TyG-FI | 3.98 (3.01 ~ 5.25) | <.001 |  | 4.19 (3.06 ~ 5.73) | <.001 |  | 4.08 (2.96 ~ 5.62) | <.001 |  | 4.23 (3.02 ~ 5.92) | <.001 |
| TyG-FI tertiles |  |  |  |  |  |  |  |  |  |  |  |
| T1 | 1.00 (Reference) |  |  | 1.00 (Reference) |  |  | 1.00 (Reference) |  |  | 1.00 (Reference) |  |
| T2 | 9.15 (5.05 ~ 16.58) | <.001 |  | 9.32 (5.18 ~ 16.77) | <.001 |  | 9.23 (5.12 ~ 16.64) | <.001 |  | 8.95 (4.82 ~ 16.62) | <.001 |
| T3 | 22.95 (13.02 ~ 40.44) | <.001 |  | 24.29 (13.56 ~ 43.49) | <.001 |  | 22.89 (12.81 ~ 40.88) | <.001 |  | 20.56 (10.96 ~ 38.59) | <.001 |
| OR: Odds Ratio, CI: Confidence Interval | | | | | | | | | | | |
| Model1: Crude | | | | | | | | | | | |
| Model2: Adjust: Adjust for the following confounding factors: age, gender. | | | | | | | | | | | |
| Model3: Adjust: Adjust for the following confounding factors: age, gender, race, education level, marital status. | | | | | | | | | | | |
| Model4: Adjust: Adjust for the following confounding factors: age, gender, race, education level, marital status, smoking status, alcohol consumption, BMI, hypertension, diabetes, eGFR. | | | | | | | | | | | |
