# Supplementary material for: Comparison analysis of the triglyceride-glucose index, triglyceride-glucose and frailty index in cardiovascular disease and mortality association among patients with rheumatoid arthritis: A population-based cohort study
Source: Medicine (Baltimore). 2026 Oct 16;105(42):e51143. doi: 10.1097/MD.0000000000051143 (PMC13681120; doi:10.1097/MD.0000000000051143)
Supplement: Supplementary file 4 [file medi-105-e51143-s004.docx]

**Table S3.** The association between the TyG-FI index and cardiovascular disease mortality after processing the missing covariate data by imputation.

| Variables | Model1 | |  | Model2 | |  | Model3 | |  | Model4 | |
| --- | --- | --- | --- | --- | --- | --- | --- | --- | --- | --- | --- |
|  | HR (95%CI) | *P* |  | HR (95%CI) | *P* |  | HR (95%CI) | *P* |  | HR (95%CI) | *P* |
| TyG-FI | 2.38 (1.82 - 3.10) | <.001 |  | 2.39 (1.73 - 3.30) | <.001 |  | 2.27 (1.65 - 3.14) | <.001 |  | 1.95 (1.29 - 2.94) | 0.002 |
| TyG-FI tertiles |  |  |  |  |  |  |  |  |  |  |  |
| T1 | 1.00 (Reference) |  |  | 1.00 (Reference) |  |  | 1.00 (Reference) |  |  | 1.00 (Reference) |  |
| T2 | 2.46 (1.08 - 5.60) | 0.032 |  | 2.49 (1.18 - 5.27) | 0.017 |  | 2.84 (1.30 - 6.23) | 0.009 |  | 2.87 (1.13 - 7.29) | 0.027 |
| T3 | 5.66 (2.38 - 13.49) | <.001 |  | 6.11 (2.70 - 13.84) | <.001 |  | 6.65 (2.97 - 14.86) | <.001 |  | 5.05 (1.78 - 14.32) | 0.002 |
| HR: Hazard Ratio, CI: Confidence Interval | | | | | | | | | | | |
| Model1: Crude | | | | | | | | | | | |
| Model2: Adjust: Adjust for the following confounding factors: age, gender. | | | | | | | | | | | |
| Model3: Adjust: Adjust for the following confounding factors: age, gender, race, education level, marital status. | | | | | | | | | | | |
| Model4: Adjust: Adjust for the following confounding factors: age, gender, race, education level, marital status, smoking status, alcohol consumption, BMI, hypertension, diabetes, eGFR. | | | | | | | | | | | |
