# Supplementary material for: Comparison analysis of the triglyceride-glucose index, triglyceride-glucose and frailty index in cardiovascular disease and mortality association among patients with rheumatoid arthritis: A population-based cohort study
Source: Medicine (Baltimore). 2026 Oct 16;105(42):e51143. doi: 10.1097/MD.0000000000051143 (PMC13681120; doi:10.1097/MD.0000000000051143)
Supplement: Supplementary file 5 [file medi-105-e51143-s005.docx]

**Table S6.** The association between the TyG-FI index and cardiovascular disease with additional adjustment for physical activity,energy intake and PIR.

| Variables | Model1 | |  | Model2 | |  | Model3 | |  | Model4 | |
| --- | --- | --- | --- | --- | --- | --- | --- | --- | --- | --- | --- |
|  | OR (95%CI) | *P* |  | OR (95%CI) | *P* |  | OR (95%CI) | *P* |  | OR (95%CI) | *P* |
| TyG-FI | 3.36 (2.64–4.28) | <.001 |  | 3.62 (2.80–4.68) | <.001 |  | 3.59 (2.75–4.70) | <.001 |  | 3.92 (2.79–5.52) | <.001 |
| TyG-FI tertiles |  |  |  |  |  |  |  |  |  |  |  |
| T1 | 1.00 (Reference) |  |  | 1.00 (Reference) |  |  | 1.00 (Reference) |  |  | 1.00 (Reference) |  |
| T2 | 6.78 (3.57–12.89) | <.001 |  | 6.78 (3.54–12.99) | <.001 |  | 7.08 (3.68–13.64) | <.001 |  | 7.55 (3.74–15.21) | <.001 |
| T3 | 15.17 (8.12–28.33) | <.001 |  | 15.41 (8.16–29.11) | <.001 |  | 15.18 (7.97–28.91) | <.001 |  | 15.68 (7.44–33.07) | <.001 |
| OR: Odds Ratio, CI: Confidence Interval | | | | | | | | | | | |
| Model1: Crude | | | | | | | | | | | |
| Model2: Adjust: Adjust for the following confounding factors: age, gender. | | | | | | | | | | | |
| Model3: Adjust: Adjust for the following confounding factors: age, gender, race, education level, marital status. | | | | | | | | | | | |
| Model4: Adjust: Adjust for the following confounding factors: age, gender, race, education level, marital status, smoking status, alcohol consumption, BMI, hypertension, diabetes, eGFR, energy intake, sport, PIR. | | | | | | | | | | | |
