# Supplementary material for: Comparison analysis of the triglyceride-glucose index, triglyceride-glucose and frailty index in cardiovascular disease and mortality association among patients with rheumatoid arthritis: A population-based cohort study
Source: Medicine (Baltimore). 2026 Oct 16;105(42):e51143. doi: 10.1097/MD.0000000000051143 (PMC13681120; doi:10.1097/MD.0000000000051143)
Supplement: Supplementary file 6 [file medi-105-e51143-s006.docx]

**Table S7.** The association between the TyG-FI index and cardiovascular disease mortality with additional adjustment for physical activity,energy intake and PIR.

| Variables | Model1 | |  | Model2 | |  | Model3 | |  | Model4 | |
| --- | --- | --- | --- | --- | --- | --- | --- | --- | --- | --- | --- |
|  | HR (95%CI) | *P* |  | HR (95%CI) | *P* |  | HR (95%CI) | *P* |  | HR (95%CI) | *P* |
| TyG-FI | 2.01 (1.51–2.68) | <.001 |  | 2.47 (1.75–3.48) | <.001 |  | 2.43 (1.70–3.48) | <.001 |  | 2.07 (1.29–3.32) | 0.003 |
| TyG-FI tertiles |  |  |  |  |  |  |  |  |  |  |  |
| T1 | 1.00 (Reference) |  |  | 1.00 (Reference) |  |  | 1.00 (Reference) |  |  | 1.00 (Reference) |  |
| T2 | 3.70 (1.48–9.22) | 0.005 |  | 4.30 (1.70–10.88) | 0.002 |  | 4.43 (1.69–11.59) | 0.002 |  | 3.96 (1.43–10.97) | 0.008 |
| T3 | 5.11 (2.11–12.40) | <.001 |  | 5.47 (2.20–13.57) | <.001 |  | 5.94 (2.30–15.35) | <.001 |  | 3.52 (1.17–10.61) | 0.026 |
| HR: Hazard Ratio, CI: Confidence Interval | | | | | | | | | | | |
| Model1: Crude | | | | | | | | | | | |
| Model2: Adjust: Adjust for the following confounding factors: age, gender. | | | | | | | | | | | |
| Model3: Adjust: Adjust for the following confounding factors: age, gender, race, education level, marital status. | | | | | | | | | | | |
| Model4: Adjust: Adjust for the following confounding factors: age, gender, race, education level, marital status, smoking status, alcohol consumption, BMI, hypertension, diabetes, eGFR, energy intake, sport, PIR. | | | | | | | | | | | |
