# Supplementary material for: Comparison analysis of the triglyceride-glucose index, triglyceride-glucose and frailty index in cardiovascular disease and mortality association among patients with rheumatoid arthritis: A population-based cohort study
Source: Medicine (Baltimore). 2026 Oct 16;105(42):e51143. doi: 10.1097/MD.0000000000051143 (PMC13681120; doi:10.1097/MD.0000000000051143)
Supplement: Supplementary file 7 [file medi-105-e51143-s007.docx]

**Table S4.** The association between TyG-FI and mortality from cardiovascular disease based on competitive risk model.

| Variables | Model1 | |  | Model2 | |  | Model3 | |  | Model4 | |
| --- | --- | --- | --- | --- | --- | --- | --- | --- | --- | --- | --- |
|  | SHR (95%CI) | *P* |  | SHR (95%CI) | *P* |  | SHR (95%CI) | *P* |  | SHR (95%CI) | *P* |
| TyG-FI tertiles |  |  |  |  |  |  |  |  |  |  |  |
| T1 | 1.00 (Reference) |  |  | 1.00 (Reference) |  |  | 1.00 (Reference) |  |  | 1.00 (Reference) |  |
| T2 | 2.14 (0.99 - 4.65) | 0.054 |  | 3.16 (1.20 - 8.33) | 0.020 |  | 4.25 (1.48 - 12.16) | 0.007 |  | 4.41 (1.21 - 16.12) | 0.025 |
| T3 | 2.30 (1.06 - 5.00) | 0.035 |  | 4.52 (1.67 - 12.19) | 0.003 |  | 5.70 (2.01 - 16.20) | 0.001 |  | 3.79 (1.01 - 14.23) | 0.049 |
| SHR:Subdistribution Hazard Ratio, CI: Confidence Interval | | | | | | | | | | | |
| Model1: Crude | | | | | | | | | | | |
| Model2: Adjust: Adjust for the following confounding factors: age, gender. | | | | | | | | | | | |
| Model3: Adjust: Adjust for the following confounding factors: age, gender, race, education level, marital status. | | | | | | | | | | | |
| Model4: Adjust: Adjust for the following confounding factors: age, gender, race, education level, marital status, smoking status, alcohol consumption, BMI, hypertension, diabetes, eGFR. | | | | | | | | | | | |
